# Role of Nuclear Factor (Erythroid-Derived 2)-Like 2 Signaling for Effects of Fumaric Acid Esters on Dendritic Cells

**DOI:** 10.3389/fimmu.2017.01922

**Published:** 2017-12-22

**Authors:** Anna Hammer, Anne Waschbisch, Ilka Knippertz, Elisabeth Zinser, Johannes Berg, Stefanie Jörg, Kristina Kuhbandner, Christina David, Jingbo Pi, Antonios Bayas, De-Hyung Lee, Aiden Haghikia, Ralf Gold, Alexander Steinkasserer, Ralf A. Linker

**Affiliations:** ^1^Department of Neurology, University Hospital Erlangen, Friedrich-Alexander-University Erlangen-Nürnberg, Erlangen, Germany; ^2^Department of Immune Modulation, University Hospital Erlangen, Friedrich-Alexander-University Erlangen-Nürnberg, Erlangen, Germany; ^3^Department of Dermatology, University Hospital Erlangen, Friedrich-Alexander-University Erlangen-Nürnberg, Erlangen, Germany; ^4^Department of Neurology, Ruhr-University Bochum, Bochum, Germany; ^5^School of Public Health, China Medical University, Shenyang, China; ^6^Department of Neurology, Hospital Augsburg, Augsburg, Germany

**Keywords:** nuclear factor (erythroid-derived 2)-like 2/antioxidant response element signaling pathway, dendritic cells, inflammation, experimental autoimmune encephalomyelitis/multiple sclerosis, oxidative stress, antigen-presenting cells

## Abstract

To date, the intracellular signaling pathways involved in dendritic cell (DC) function are poorly understood. The antioxidative transcription factor nuclear factor (erythroid-derived 2)-like 2 (Nrf2) has been shown to affect maturation, function, and subsequent DC-mediated T cell responses of murine and human DCs. In experimental autoimmune encephalomyelitis (EAE), as prototype animal model for a T helper cell-mediated autoimmune disease, antigen presentation, cytokine production, and costimulation by DCs play a major role. We explore the role of Nrf2 in DC function, and DC-mediated T cell responses during T cell-mediated autoimmunity of the central nervous system using genetic ablation and pharmacological activation in mice and men to corroborate our data in a translational setting. In murine and human DCs, monomethyl fumarate induced Nrf2 signaling inhibits DC maturation and DC-mediated T cell proliferation by reducing inflammatory cytokine production and expression of costimulatory molecules. In contrast, Nrf2-deficient DCs generate more activated T helper cells (Th1/Th17) but fewer regulatory T cells and foster T cell proliferation. Transfer of DCs with Nrf2 activation during active EAE reduces disease severity and T cell infiltration. Our data demonstrate that Nrf2 signaling modulates autoimmunity in murine and human systems *via* inhibiting DC maturation and function thus shedding further light on the mechanism of action of antioxidative stress pathways in antigen-presenting cells.

## Introduction

In the periphery, immature dendritic cells (DCs) survey their environment and capture soluble antigens, like microbes and cell debris. Since they are barely immunogenic, immature DC express only low levels of major histocompatibility complex (MHC) II and costimulatory molecules but display marked phagocytic capacity which renders them critical for maintaining peripheral T cell tolerance (1, 2). Activation of DC leads to maturation with increased surface marker expression and cytokine production allowing them to efficiently present antigens to naive T cells (3, 4). Consequently, DC subsets and functions are altered in many autoimmune diseases including multiple sclerosis (MS), systemic lupus erythematosus or graft-versus-host disease (5–7). While DC maturation and corresponding changes in surface marker expression are well characterized (8), the involved intracellular signaling pathways are less understood.

Recently, the antioxidant transcription factor nuclear factor (erythroid-derived 2)-like 2 (Nrf2) was described as critical regulator responsible for maintenance of DC redox homeostasis, differentiation, and maturation by modulating the expression of cytoprotective genes, such as heme oxygenase 1 (Hmox1), NAD(P)H: quinone oxidoreductase 1 (Nqo1), and glutathione (9–12). In general, Nrf2 is involved in different aspects of cellular functions including differentiation, proliferation and inflammation by fostering antioxidative gene expression while suppressing the production of reactive oxygen species and proinflammatory cytokines as well as nuclear factor kappa-light-chain-enhancer of activated B cells (NF-κB) signaling (13–17). Furthermore, aberrant function or expression of Nrf2 is associated with pathologies such as inflammatory diseases, autoimmunity, cancer and neurodegeneration [as reviewed in Ref. (18, 19)]. In the cytosol, the activity of Nrf2 is tightly regulated *via* proteasomal degradation by Kelch-like ECH-associated protein 1 (Keap1) (20, 21). Appropriate stimuli, like oxidative stress, induce conformational changes in Keap1 allowing for translocation of Nrf2 into the nucleus where it binds to antioxidant response elements (ARE) and activates gene expression (21). Nrf2-deficient mice only tolerate mild autoimmune attacks, and if so they display an aggravated disease course in several inflammation-mediated animal models (22, 23), including experimental autoimmune encephalomyelitis (EAE) (24). In contrast, systemic activation of Nrf2 mitigates autoimmune inflammation in scurfy mice (25).

Fumaric acid esters, like dimethyl fumarate (DMF) and its primary active metabolite monomethyl fumarate (MMF), have been shown to activate the Nrf2-ARE pathway by altering the ability of Keap1 to interact with Nrf2 (26) and reduce DC maturation, cytokine release and T cell activation capacities mainly *via* NF-κB signaling pathways *in vitro* (27–31). In EAE, treatment with DMF led to significant therapeutic effects on parameters of inflammation and neurodegeneration (26, 32). Similar observations were made in MS clinical trials (33, 34). Thus, MMF or DMF treatment can be employed as an excellent model system to analyze effects of Nrf2 activation in DC.

Here, we focus on the effect of Nrf2 signaling on DC maturation, function and subsequent T cell responses *in vitro* as well as in T cell-mediated autoimmunity of the central nervous system *in vivo*. We show that Nrf2 deficiency fosters a proinflammatory phenotype in murine and human DC, while Nrf2 activation promotes rather tolerogenic functions by significantly modulating cytokine production and expression of costimulatory molecules.

## Materials and Methods

### Mice

Nuclear factor (erythroid-derived 2)-like 2-fl/fl mice were a kind gift from Prof. Pi, China Medical University, Shenyang, China. CD11c-Cre mice were obtained from Prof. Steinkasserer, University of Erlangen-Nürnberg, Erlangen, Germany. Mice harboring a transgenic MOG-specific T cell receptor [2D2 mice (35)]; were a kind gift from Prof. V. Kuchroo, Boston, MA, USA. Wild-type (wt), 2D2, Nrf2KO, and CD11c-Cre/Nrf2-fl/fl mice were backcrossed on the C57BL/6J background and all mice were obtained from an in-house breed at the local animal care facility of the University of Erlangen-Nürnberg or the Ruhr-University Bochum under a 12-h day–night cycle and standardized environment. All experiments were performed in accordance with the German laws for animal protection and were approved by local ethic committees (Government of Unterfranken, Bavaria, Germany, ref. # 55.2-2532-2-451).

### Oral DMF Gavage and EAE Induction

Mice received 15 mg/kg body weight DMF (Sigma-Aldrich, St. Louis, MO, USA) in 0.8 mg/ml hydroxypropyl methylcellulose (Sigma-Aldrich) twice daily by oral gavage, while control mice obtained 0.8 mg/ml hydroxypropyl methylcellulose. Mice were adapted to DMF treatment 2 weeks before EAE induction. Since the majority of DMF is rapidly metabolized to MMF by esterases in the intestine and only MMF can be detected in the systemic circulation (36, 37), we employed DMF to examine effects of Nrf2 pathway activation in our *in vivo* studies but MMF for *in vitro* stimulation experiments.

Induction of active EAE was performed as previously described (38, 39). For DC transfer experiments, 1 × 10^5^ DC, cultured *in vitro* for 10 days with or without 200 µM MMF (Sigma-Aldrich) and stimulated with 1 µg/ml lipopolysaccharide (LPS) (Sigma-Aldrich) for 24 h, were injected intraperitoneally at d2 and d7 after EAE induction together with 200 ng pertussis toxin (List Biological Laboratories).

### Generation of Murine Bone Marrow-Derived DC (BMDC)

Bone marrow cells were obtained from tibias and femurs and maintained in R10 medium (RPMI-1640 + 10% FCS + 1% penicillin/streptomycin + 1% l-glutamine) containing granulocyte macrophage colony-stimulating factor (GM-CSF) (5 ng/ml, Biolegend, San Diego, CA, USA) and interleukin (IL)-4 (5 ng/ml, Biolegend) for 10 days with/without 200 µM MMF (Sigma-Aldrich). Cells were matured on day 8 with 1 µg/ml LPS (Sigma-Aldrich). For flow cytometry, cells were stained with αCD11c (N418, Biolegend), αCD80 (16-10A1, Biolegend), αCD86 (GL-1, Biolegend), and αMHCII (AF6-120.1, BD Biosciences, San Diego, CA, USA).

### DC Functional Assays

Phagocytosis was assessed by stimulating DC with 1 µg/ml LPS (Sigma-Aldrich) for 48 h before adding opsonized Fluoresbrite^®^ YG Carboxylate Microspheres 0.75 µm (Polysciences Inc., Warrington, PA, USA). After 20 h of incubation, cells were subjected to flow cytometry and phagocytic cells were detected in the FITC channel.

The migratory capacity of DC was investigated using a modified Boyden chamber assay and transwell inserts with a 5 µm porous bottom (Corning Life Sciences, Corning, NY, USA). Mature DC were seeded into the migration chamber in medium containing 0.5% FCS. The lower chamber was supplemented with 10% FCS medium. After 20 h of migration, cells residing in the lower chamber (transmigrated fraction) were stained for CD11c and quantified by flow cytometry (FACSCantoII, BD Biosciences).

### Murine Coculture Assays

Mature DC were loaded with MOG_35–55_ peptide (20 µg/ml) for 1 h and seeded with naive transgenic T cells harboring a MOG specific TCR at a ratio of 1:6. Naive MOG-specific transgenic T cells were isolated form the spleen of 2D2 mice by magnetic activated cell sorting using the “Naive CD4^+^ T Cell Isolation Kit, mouse” according to the manufacturer’s instructions (Miltenyi Biotech, Bergisch Gladbach, Germany). For Th17 differentiations, cocultured cells were additionally stimulated with IL-6 (40 ng/ml, R&D Systems, Minneapolis, MN, USA) and transforming growth factor beta (TGFβ1) (2 ng/ml, Biolegend). For Th1 differentiations, cells were cultured in the presence of IL-12p70 (20 ng/ml, R&D Systems) and anti-IL-4 (10 mg/ml, 11B11, Biolegend). For Treg differentiations, TGFβ1 (10 ng/ml, Biolegend) was added to culture media. T cell frequencies were analyzed by flow cytometry (FACSCantoII, BD Biosciences) using the antibodies listed in the section “Murine FACS analysis.” To monitor proliferation, cells were pulsed with [3H]-thymidine 24 h before analysis.

### Murine T Cell Differentiation

Differentiation of murine T cells was performed as previously described (40). Splenic T cells were isolated by magnetic activated cell sorting using the “pan T cell isolation kit II” according to the manufacturer’s instructions (Miltenyi Biotech). Cells were fluorescently stained with an antibody cocktail containing αCD4-FITC (RM4-5, eBioscience), αCD44-PE (IM7, Biolegend), αCD62L-APC (MEL-14, eBioscience), and αCD25-PE-Cy5 (PC61.5, eBioscience) and were subsequently isolated by fluorescence activated cell sorting on MoFlo (Beckman-Coulter, Brea, CA, USA) in the FACS-core unit of the University Hospital Erlangen. Sorted naive T cells (CD4^+^CD62L^+^CD44^low^CD25^−^) were stimulated by plate-bound anti-CD3 (2 µg/ml, 145-2C11, BD Pharmingen, San Diego, CA, USA) and soluble anti-CD28 (2 µg/ml, 37.51, BD Pharmingen) and cultured for 4 days in the presence of IL-6 (40 ng/ml, R&D Systems, Minneapolis, MN, USA) and TGFβ1 (2 ng/ml, Biolegend) for Th17, IL-12p70 (20 ng/ml,R&D Systems) and anti-IL-4 (10 µg/ml, Biolegend) for Th1 or TGFβ1 (10 ng/ml, Biolegend) alone for Treg differentiation. T cell frequencies were analyzed by flow cytometry (FACSCantoII, BD Biosciences) using the antibodies listed in the section “Murine FACS analysis.”

### Isolation of Spinal Cord Lymphocytes

Spinal cords were disrupted with a 5 ml glass homogenizer followed by Percoll (GE Healthcare) density gradient centrifugation. Cell frequencies were analyzed by flow cytometry (FACSCantoII, BD Biosciences) using the antibodies listed in the section “Murine FACS analysis.”

### Murine FACS Analyses

As previously described (40), dead cells were excluded by the fixable viability dye eFluor^®^780 (0.2 μl/test, eBioscience). Nonspecific Fc-mediated interactions were blocked by addition of 0.5 µl αCD16/32 (93, eBioscience). For surface staining, cells were treated with the respective fluorochrome-conjugated antibodies: αCD4-BV510 (RM4-5, Biolegend), αCD11c-FITC (HL3, BD Biosciences), αCD25-APC (PC61.5, eBioscience), αCD80-APC (16-10A1, Biolegend), αCD86-PerCP/Cy5.5 (GL-1, Biolegend), and αMHCII-BV510 (M5/114.15.2, Biole-gend).

For intracellular cytokine staining, cells were stimulated for 4 h with ionomycin (1 µM, Sigma-Aldrich) and PMA (50 ng/ml, Sigma-Aldrich) in the presence of monensin (2 µM, eBioscience), fixed with 1% paraformaldehyde and made permeable by saponin buffer treatment. Intracellular cytokines were stained with the respective fluorochrome-conjugated antibodies: αFoxp3-PE (FJK-16s, eBioscience), αIFNγ-APC (XMG1.2, eBioscience), and αIL-17A-PE (eBio17B7, eBioscience). Samples were measured with a flow cytometer (FACSCantoII, BD Biosciences).

### Generation of Human DCs

For the generation of monocyte-derived DCs from leukoreduction system chambers of healthy donors (HDs) the positive vote from the local ethics committee has been obtained (Re.-Nr.: 4556). Generation of human monocyte-derived DC was performed as previously described (41). Peripheral blood mononuclear cells were prepared from leukoreduction system chambers of HDs by density centrifugation, followed by plastic adherence on tissue culture dishes (BD Falcon, San Diego, CA, USA). The non-adherent fraction was cryopreserved at −80°C for isolation of allogeneic T cells as described before (42), while the adherent cell fraction was cultured for 4 days with or without 50 or 200 µM MMF in DC-medium consisting of RPMI 1640 (Lonza, Basel, Switzerland) supplemented with 1% of heat-inactivated human serum type AB (Sigma-Aldrich), 1% Penicillin/Streptomycin/l-glutamine (Sigma-Aldrich), and 10 mM Hepes (Lonza) as well as 800 IU/ml (day 0) or 400 IU/ml (day 3) recombinant human GM-CSF and 250 IU/ml (days 0 and 3) recombinant IL-4 (both Miltenyi). Maturation of DC was induced by adding a maturation cocktail consisting of 200 U/ml IL-1β, 1,000 U/ml IL-6 (both CellGenix, Freiburg, Germany), 10 ng/ml tumor necrosis factor alpha (TNF-α) (Beromun, Boehringer Ingelheim, Germany), and 1 µg/ml PGE2 (Prostin E2, Pfizer, NJ, NY, USA) for 48 h. DC were analyzed by flow cytometry for activation marker expression: αCD80-PE (2D10.4, eBioscience), αMHCII-PE/Cy5 (LN3, eBioscience), αCD25-FITC (M-A251, BD Biosciences), αCD86-PE (IT2.2, eBioscience), αMHCI-FITC (W6/32, eBioscience), αCCR7-APC (3D12, eBioscience), αILT-4-FITC (27D6, eBioscience), and αCD83-PE (HB15e, eBioscience).

### Human Mixed Lymphocyte Reaction (MLR)

Mature human DC were generated as described above (41) and were either left untreated or were stimulated with maturation cocktail for 24 h. Allogeneic T cells were generated from cryopreserved non-adherent fraction. For this purpose, frozen cells were thawed, filtrated using a cell-strainer (Falcon) to remove dead cells and finally adjusted to 2 × 10^6^ cells/ml in MLR medium consisting of RPMI 1640 (Lonza) supplemented with 5% heat-inactivated human serum type AB (Sigma-Aldrich), 1% Penicillin/Streptomycin/l-glutamine (Sigma-Aldrich), and 10 mM HEPES (Lonza). Afterward, triplicates of 2 × 10^5^ allogeneic T cells were cocultured in 96-well flat cell culture plates (Falcon) with allogeneic DCs at different ratios for 72 h in MLR medium, and were finally pulsed with [3H]-thymidine (1 μC/well) for 8–16 h. Cultures were harvested onto glassfiber filtermates using an ICH-110 harvester (Inotech, Dottikon, Switzerland) and filters were counted in a 1450 microplate counter (Wallac, Turku, Finland).

### Cytometric Bead Array

Determination of cytokines (IL-6, TNF-α) secreted by human DC and/or T cells into the supernatant was assessed by Human Inflammation LEGENDplex™ (13-plex, Biolegend) according to the manufacturer’s protocol.

### Enzyme-Linked Immunosorbent Assay (ELISA)

Cytokine concentrations in murine cell culture supernatants were analyzed by ELISA for the secretion of IL-6, TNF-α, IL-23, and IL-12 (R&D Systems) according to the manufacturer’s instructions.

### Real-time PCR

RNA isolation, reverse transcription and PCR reactions were performed as previously described (40). Relative quantification was performed by the ΔΔCT method, normalizing target gene expression on *actb*/β-Actin or *gapdh* as housekeeping gene. The following TaqMan^®^ real-time PCR assays from Thermo Fisher Scientific were used: actb (β-Actin) Mm00607939_s1, nfe2l2 (nrf2) Mm00477784_m1, il6 Mm99999064_m1, nqo1 Mm01253561_m1, hmox1 Mm00516005_m1, il10 Mm01288386_m1, il12a Mm00434169_m1, arg1 Mm00475988_m1, tgfb Mm01178819_m1, nqo1 Hs02512143_s1, akr1c1 Hs04230636_sH, hcar2 Hs02341584_s1, and gapdh Hs02758991_g1.

### Western Blot

Cultured cells were harvested in 1× Ripa lysis buffer [15 mM NaCl, 3.85 mM SDS, 5 mM Tris, 13.4 mM SDOX, 0.05 mM EDTA, 0.1% NP40, complete protease inhibitor cocktail Complete Mini and phosphatase inhibitor cocktail PhosStop (Roche Diagnostics, Basel, Switzerland)]. Protein concentration was determined using the BC Assay Protein Quantitation Kit (Interchim, Montluçon, France). Protein was detected using a rabbit polyclonal Nqo1 antibody (1:1,000, ab34173, Abcam, Cambridge, United Kingdom). Mouse anti-β-Actin (1:1,000) was obtained from Abcam (ab8226). Donkey antirabbit Alexa Fluor 488-conjugated and goat antimouse Alexa Fluor 647-conjugated secondary antibodies (1:1,000, Invitrogen, Carlsbad, CA, USA) were used. Detection was performed using a Fusion Capt Advance FX7 (peqlab, Erlangen, Germany).

### Statistical Analysis

Statistical testing was performed using GraphPad Prism (GraphPad Software Inc., San Diego, CA, USA). All *in vitro* and *ex vivo* data were analyzed by either one-/two-way ANOVA followed by Tukey’s posttest, unpaired *t*-test or Mann–Whitney test after checking for normal distribution (unless otherwise indicated). EAE data were analyzed by Mann–Whitney *U*-test. Data are presented as mean ± SEM; *p < 0.05, **p < 0.01, or ***p < 0.001 were considered to be statistically significant.

## Results

### Nrf2 Signaling Alters DC Activation and Maturation *In Vitro*

**Figure 1:**
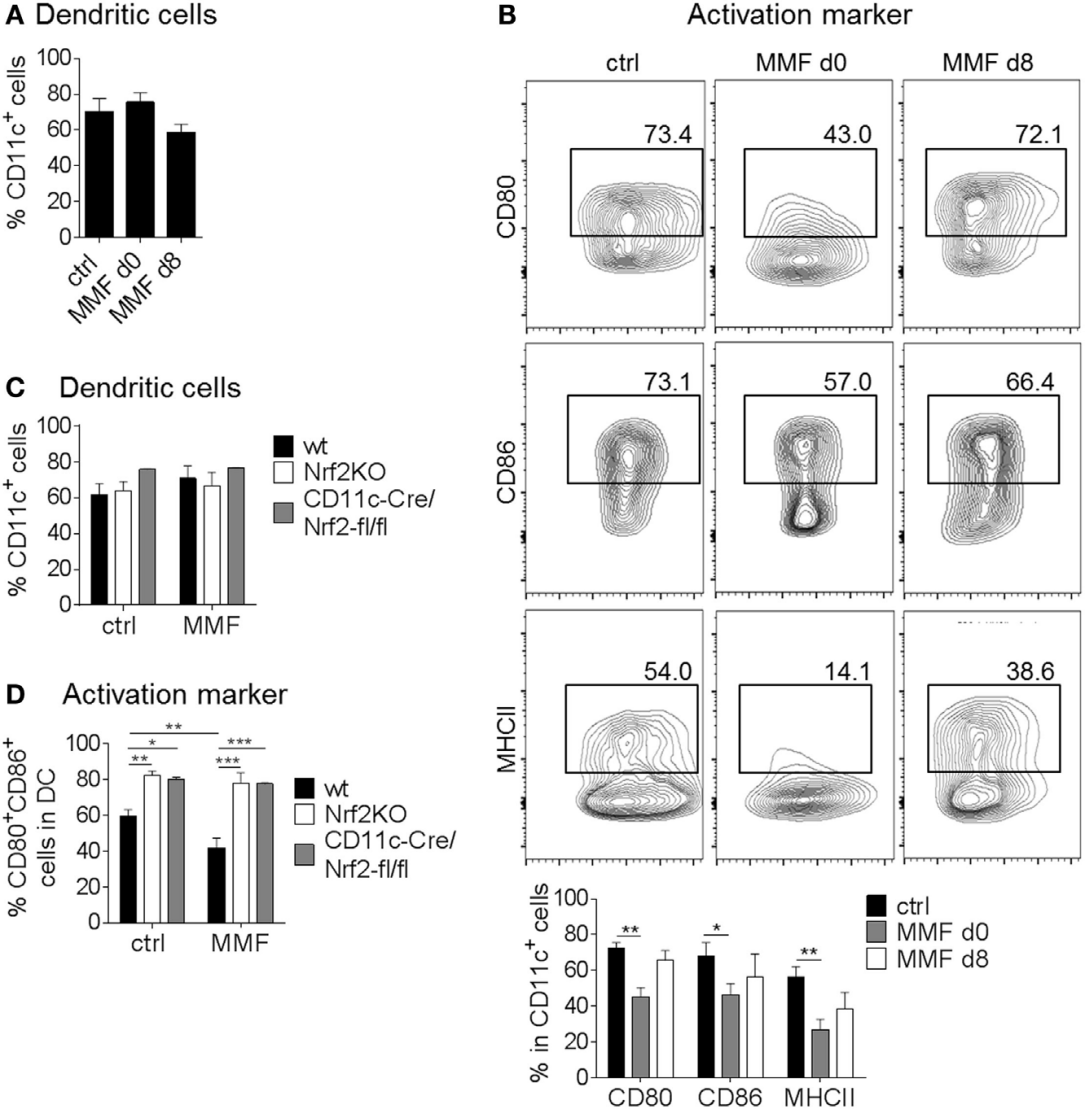
Nuclear factor (erythroid-derived 2)-like 2 (Nrf2) signaling alters dendritic cell (DC) activation status. **(A)** Frequency of CD11c^+^ cells after generating (d0) or just activating (d8) bone marrow-derived DC (BMDC) in the presence of 200 µM metabolite monomethyl fumarate (MMF) (data pooled from three to five experiments). **(B)** Expression of CD80, CD86, and MHCII on cultured CD11c^+^ BMDC after MMF treatment as assessed by flow cytometry (data pooled from three to four experiments, *p < 0.05, **p < 0.01). **(C)** Frequency of CD11c^+^ cells after generating wild-type (wt), Nrf2KO, and CD11c-Cre/Nrf2-fl/fl BMDC in the presence of 200 µM MMF (data pooled from four independent experiments). **(D)** Frequency of CD80^+^CD86^+^ cells in CD11c^+^ BMDC generated from wt, Nrf2KO, and CD11c-Cre/Nrf2-fl/fl mice under MMF treatment as assessed by flow cytometry (data pooled from four experiments, *p < 0.05, **p < 0.01, ***p < 0.001). BMDC were stimulated with 1 µg/ml LPS for 48 h before analysis. LPS stimulated, but otherwise treatment naive BMDC obtained from wt mice served as control.

To analyze Nrf2 signaling in DC, we employed Nrf2-deficient cells. In a complementary approach, MMF treatment was used to induce Nrf2 signaling in DC. BMDC were cultured with 200 µM MMF either from the beginning (day 0) or starting at the day of LPS stimulation (i.e., day 8) to analyze if the time point of Nrf2 activation is crucial for its effect on DC maturation and surface marker expression. First, we examined whether activation of Nrf2-dependent signaling pathways influences BMDC generation *in vitro*. Yet CD11c^+^ cell frequencies were not altered in MMF-treated cell cultures (Figure 1A). However, the expression of MHCII, CD80 and CD86 was reduced when BMDC were generated under Nrf2 activating conditions (day 0) whereas addition of MMF on day 8 together with LPS, had almost no effect on activation marker expression (Figure 1B; Figure S1A in Supplementary Material).

To investigate whether the effect of MMF on BMDC surface marker expression is indeed dependent on Nrf2 induced signaling pathways, we treated Nrf2-deficient BMDC generated from either complete Nrf2 knockout mice (Nrf2KO) or conditional DC specific knockout mice (CD11c-Cre/Nrf2-fl/fl) with MMF. Again, the yield of CD11c^+^ cells did not differ between bone marrow obtained from wt, Nrf2KO, or CD11c-Cre/Nrf2-fl/fl mice and treatment with 200 µM MMF did not influence the generation of BMDC in the cell culture (Figure 1C). However, flow cytometric analyses revealed enhanced expression of CD80, CD86 (Figure 1D), and CD40 (Figure S1B in Supplementary Material) on Nrf2-deficient BMDC generated from either Nrf2KO or CD11c-Cre/Nrf2-fl/fl mice compared to wt BMDC. Additionally, MMF treatment had no influence on the frequency of CD80^+^/CD86^+^ and CD80^+^/CD40^+^ Nrf2-deficient BMDC but reduced the expression of CD80, CD86, and CD40 on wt BMDC (Figure 1D; Figure S1B in Supplementary Material).

In conclusion, modulation of the Nrf2 signaling pathway critically affects the activation status of DC *in vitro*.

### Nrf2 Affects DC Functionality and Cytokine Expression Profile

**Figure 2:**
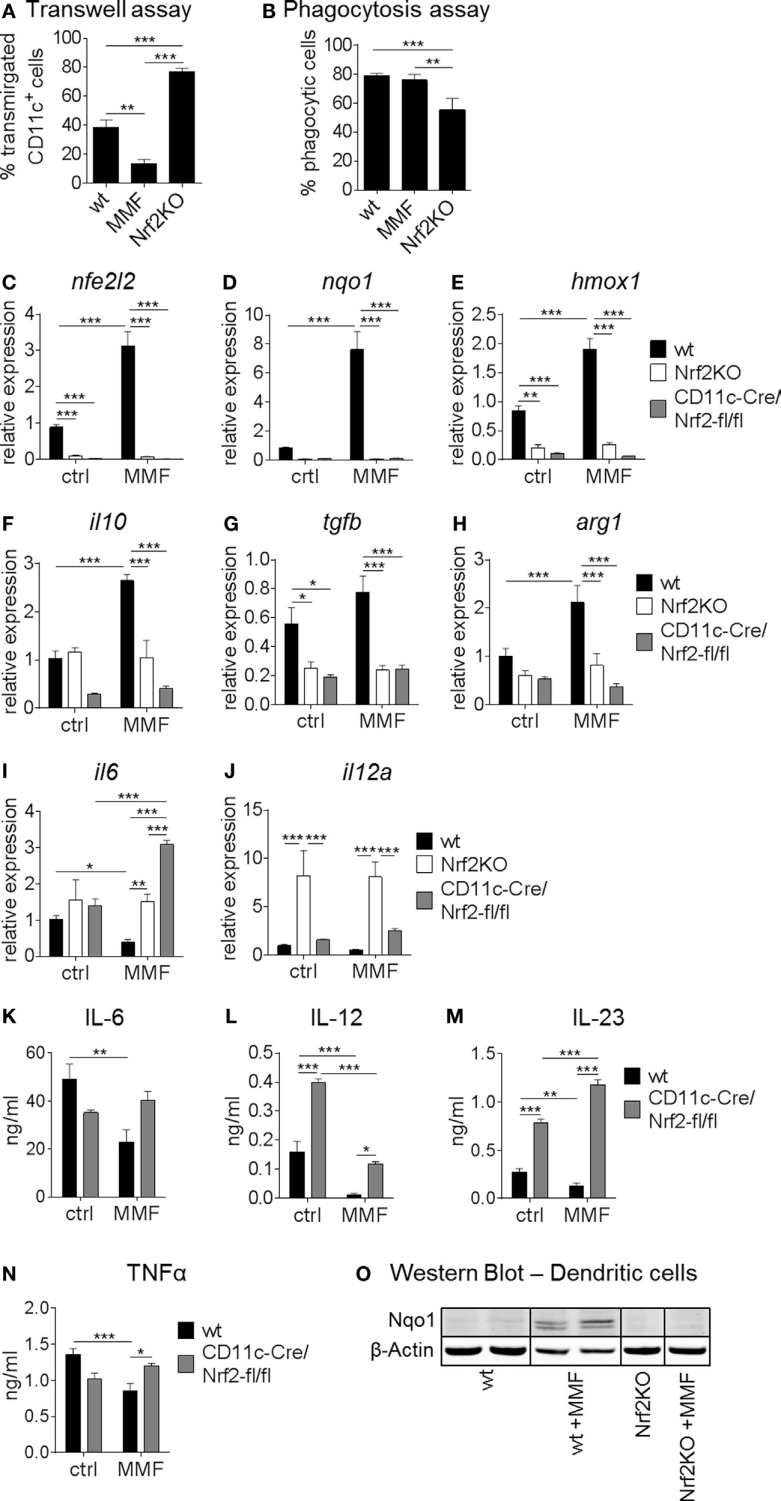
Nuclear factor (erythroid-derived 2)-like 2 (Nrf2) affects functionality of myeloid dendritic cell(DC). **(A)** Migratory capacity of Nrf2KO and metabolite monomethyl fumarate (MMF)-treated wild-type (wt) bone marrow-derived DC (BMDC) compared to control cells as assessed by an *in vitro* FCS-gradient Transwell assay (data pooled from two to five independent experiments, **p < 0.01, ***p < 0.001). **(B)** Phagocytic capacity of MMF versus control-treated wt and Nrf2KO BMDC determined as frequency of fluorescent bead uptake by flow cytometry (data pooled from two to four experiments, **p < 0.01, ***p < 0.001). **(C–J)** Gene expression analysis of *nfe2l2* (Nrf2) **(C)**, *nqo1* **(D)**, *hmox1* **(E)**, *il10* **(F)**, *tgfb* **(G)**, *arg1* **(H)**, *il6* **(I)**, and *il12a* **(J)** in MMF-treated wt and Nrf2-deficient BMDC compared to control cells (data pooled from two to three preparations, *p < 0.05, **p < 0.01, ***p < 0.001). **(K–N)** Interleukin (IL)-6 **(K)**, IL-12 **(L)**, IL-23 **(M)**, and tumor necrosis factor alpha **(N)** production in MMF-treated wt and Nrf2-deficient cultured BMDC (*n* = 6 for wt and *n* = 3 for CD11c-Cre/Nrf2-fl/fl, *p < 0.05, **p < 0.01, ***p < 0.001). **(O)** Representative immunoblot of NAD(P)H: quinone oxidoreductase 1 (Nqo1) protein expression in wt and Nrf2-deficient BMDC cultured with and without MMF. β-Actin was used as loading control.

Next, we assessed the influence of Nrf2 on different essential BMDC functions *in vitro*. As shown in Figure 2A, migratory capacities of BMDC isolated from Nrf2KO mice was significantly increased compared to BMDC from wt mice. Furthermore, MMF-treated wt BMDC showed a strongly reduced migratory capacity compared to untreated wt mice (Figure 2A). The phagocytosis rate of MMF-treated wt BMDC was not altered compared to untreated wt BMDC but decreased in Nrf2-deficient BMDC which correlates well with their enhanced maturation status (Figure 2B).

Expression analysis revealed that MMF treatment enhanced Nrf2 mRNA levels (Figure 2C) in wt BMDC *in vitro*. In turn, Nrf2 activation not only induced its antioxidative target genes *nqo1* and *hmox1* but also the anti-inflammatory cytokine *il10* (Figures 2D–F). As expected, *nfe2l2* (Nrf2), *nqo1*, and *hmox1* expression was almost undetectable in Nrf2-deficient BMDC and *tgfb* expression levels were also significantly lower compared to wt BMDC (Figures 2C–E,G; Figure S2 in Supplementary Material). Furthermore, Nrf2 activation enhanced, while Nrf2 deficiency reduced, the expression of *arginase 1 (arg1)*, a marker for tolerogenic DC (Figure 2H). Nrf2KO BMDC displayed a higher expression level of *il12a* compared to wt BMDC, while Nrf2 activation decreased proinflammatory cytokine expression on the mRNA level (Figures 2I,J). Nrf2 induction significantly reduced the production of IL-6, IL-12, IL-23, and TNF-α, proinflammatory cytokines that mediate T helper (Th)1 and Th17 differentiation, in wt but not Nrf2KO (data not shown) or CD11c-Cre/Nrf2-fl/fl BMDC (Figures 2K–N). Furthermore, Nrf2-deficient BMDC tended to produce higher baseline proinflammatory but lower tolerogenic cytokine levels compared to wt cells (Figure 2).

Immunoblot analysis of Nqo1 protein in wt and Nrf2-deficient BMDC cultures confirmed that the induction of Nqo1 expression in BMDC by MMF treatment is Nrf2 dependent (Figure 2O; Figure S3 in Supplementary Material).

Taken together, these data indicate that Nrf2 signaling alters DC functionality by modulating maturation, migration, expression of costimulatory molecules, and cytokine/chemokine production, which are required for effective antigen presentation to T cells.

### Nrf2 Directly and Indirectly Regulates T Cell Differentiation and Proliferation

**Figure 3:**
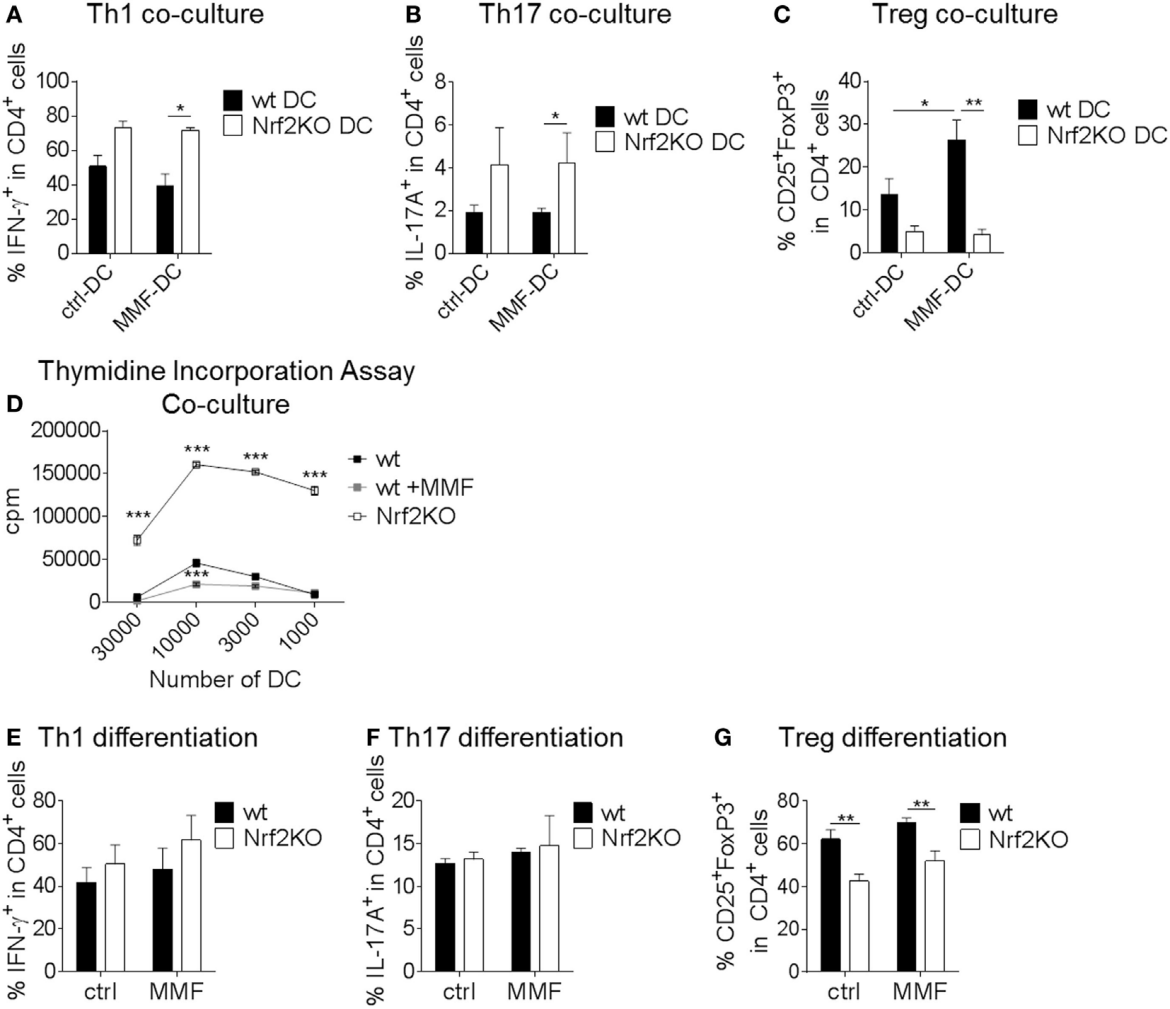
Nuclear factor (erythroid-derived 2)-like 2 (Nrf2Nrf2) shows direct and indirect effects on T cell differentiation. **(A–C)** MOG-loaded, metabolite monomethyl fumarate (MMF) pretreated or untreated wt and Nrf2KO bone marrow-derived dendritic cell (BMDC) were cocultured with MOG-specific transgenic naive CD4^+^ T cells under Th1 **(A)**, Th17 **(B)**, or Treg **(C)** cell polarizing conditions (data pooled from three to six experiments, *p < 0.05, **p < 0.01). **(D)** Proliferation rate of T cells cultured with Nrf2KO or MMF pretreated or untreated wild-type (wt) BMDC as assessed by thymidine incorporation assay (one out of three representative experiments is shown, ***p < 0.001, cpm = counts per minute). **(E–G)** Addition of 200 µM MMF to wt and Nrf2KO CD4^+^ T cell differentiation culture under Th1 **(E)**, Th17 **(F)**, or Treg **(G)** polarizing conditions (data pooled from three to five experiments, **p < 0.01).

In coculture experiments, untreated or MMF pretreated wt and Nrf2KO BMDC were cultured together with naive CD4^+^ T cells under Th1, Th17, or Treg cell polarizing conditions to demonstrate effects of Nrf2 signaling on DC-mediated T cell activation. Nrf2-deficient BMDC increased Th1 and Th17 but reduced Treg differentiation in coculture (Figures 3A–C). MMF pretreatment of wt BMDC had no effect on Th1 and Th17 frequencies (Figures 3A,B) but increased Treg differentiation (Figure 3C). Thymidine incorporation showed an enhanced proliferation rate of T cells that were cultured together with Nrf2KO BMDC and a reduced proliferation in the coculture with MMF pretreated wt BMDC (Figure 3D).

T cell monocultures demonstrated that Nrf2 had no direct effect on Th1 and Th17 cell differentiation (Figures 3E,F). However, Nrf2 deficiency induced a distinct reduction in Treg differentiation (Figure 3G).

These results show that Nrf2 signaling in DC may modulate their potential to regulate T cell differentiation and proliferation.

### Nrf2 Influences Disease Course and Immune Cell Activation in the EAE Mouse Model

To demonstrate the relevance of the Nrf2 pathway in DC function for T cell-mediated autoimmunity *in vivo*, we employed the EAE animal model.

**Figure 4:**
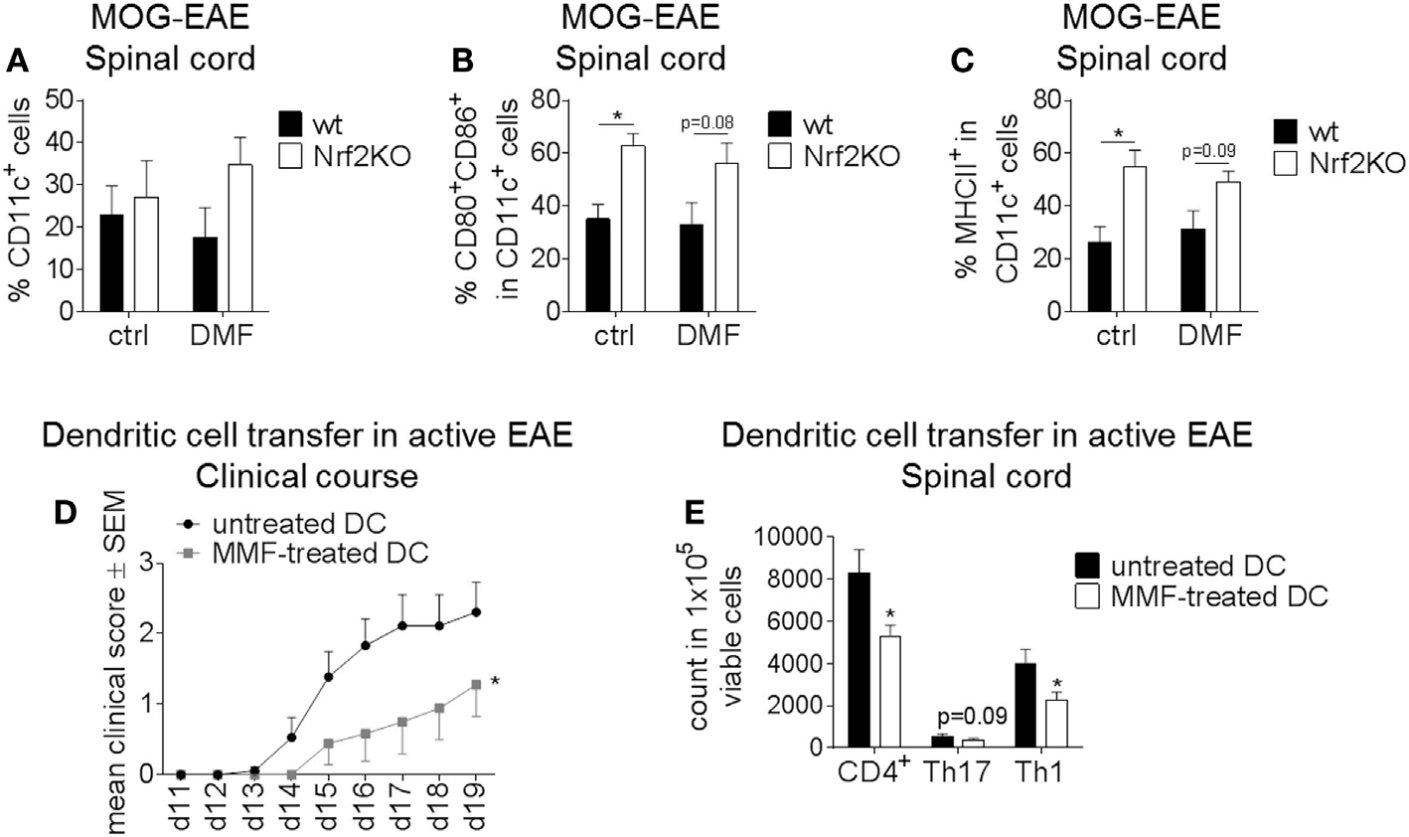
Nuclear factor (erythroid-derived 2)-like 2 (Nrf2) signaling alters disease course and immune cell activation in the experimental autoimmune encephalomyelitis (EAE) mouse model. **(A–C)** *Ex vivo* flow cytometry analysis of CD11c^+^ cells **(A)** as well as CD80/CD86 **(B)** and MHCII **(C)** expression in the spinal cord under dimethyl fumarate (DMF) versus control treatment on day 14 after active EAE induction (*n* = 3–4 mice per group, *p < 0.05). **(D)** Clinical course of MOG_35–55_ EAE after dendritic cell (DC)-transfer. Untreated or metabolite monomethyl fumarate (MMF) pretreated *in vitro* cultured bone marrow-derived DC (BMDC) were transferred into EAE mice on days 2 and 7 after MOG immunization. Data are shown on a 5-point score scale (*n* = 9 mice per group, data pooled from two independent experiments, *p < 0.05). **(E)** *Ex vivo* flow cytometry analysis of total CD4^+^, Th1, and Th17 cells in the spinal cord of DC-transferred mice at the maximum of disease (*n* = 7–8 mice per group, data pooled from two independent experiments, *p < 0.05).

Spinal cords of DMF or control-treated wt and Nrf2KO mice were analyzed for DC frequencies and surface marker expression on day 14 after MOG-EAE induction (Figures 4A–C). Neither Nrf2 activation nor deficiency had an effect on the frequency of CD11c^+^ cells in the spinal cord of EAE mice (Figure 4A). In good accordance with the previous data, Nrf2 deficiency caused a significant increase in the expression of the activation markers CD80 and CD86 and enhanced MHCII expression on CD11c^+^ DC in the spinal cord (Figures 4B,C).

To further elucidate the effect of Nrf2 modulation on DC function during neuroinflammation, we employed MMF treatment as a potent Nrf2 inducer. *In vitro* cultured untreated or MMF-treated BMDC were injected into untreated wt mice at d2 and d7 after EAE induction. Mice which received MMF-treated BMDC showed an ameliorated clinical disease course compared to the control group (Figure 4D) and also a significantly reduced amount of total CD4^+^ as well as Th1 and Th17 cells in the spinal cord (Figure 4E).

Therefore, Nrf2 induction in DC *in vitro* may promote a tolerogenic phenotype enabling these cells to mitigate active EAE *in vivo*.

### Nrf2 Signaling Alters Maturation Status, Gene Expression Profile, and T Cell Activation Capacity of Human DC *In Vitro*

**Figure 5:**
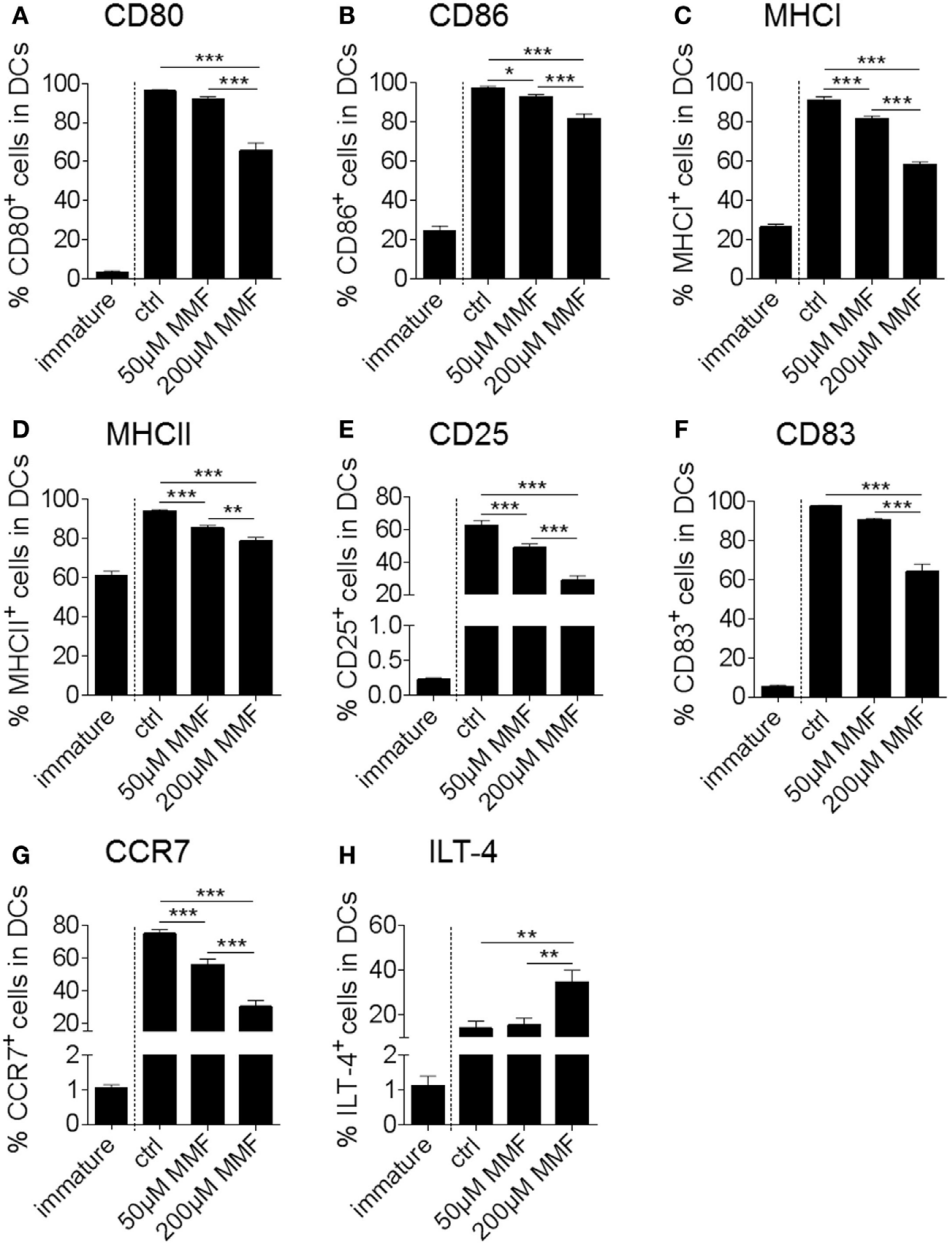
Nuclear factor (erythroid-derived 2)-like 2 (Nrf2) affects maturation marker expression of human dendritic cell (DC) *in vitro*. Frequency of CD80 **(A)**, CD86 **(B)**, MHCI **(C)**, MHCII **(D)**, CD25 **(E)**, CD83 **(F)**, CCR7 **(G)**, and ILT-4 **(H)** positive cells in total human monocyte-derived DC under metabolite monomethyl fumarate (MMF) treatment as assessed by flow cytometry. The immature control group was not treated with the maturation cocktail as described in the method section. Statistical significance was tested between the matured, untreated control group (ctrl) and the MMF-treated groups (data pooled from four to five independent experiments, *p < 0.05, **p < 0.01, ***p < 0.001).

In a next step, we translated our findings from murine to human DC. Treatment of *in vitro* cultured human monocyte-derived DC with increasing amounts of the potent Nrf2 inducer MMF caused a concentration-dependent reduction of DC costimulatory, activation and inflammatory molecules including CD80, CD86, MHCI, MHCII, CD25, CD83, and C-C chemokine receptor type 7 (CCR7) as well as an increased expression of the tolerogenic surface receptor Ig-like transcript (ILT)-4 (Figures 5A–H).

**Figure 6:**
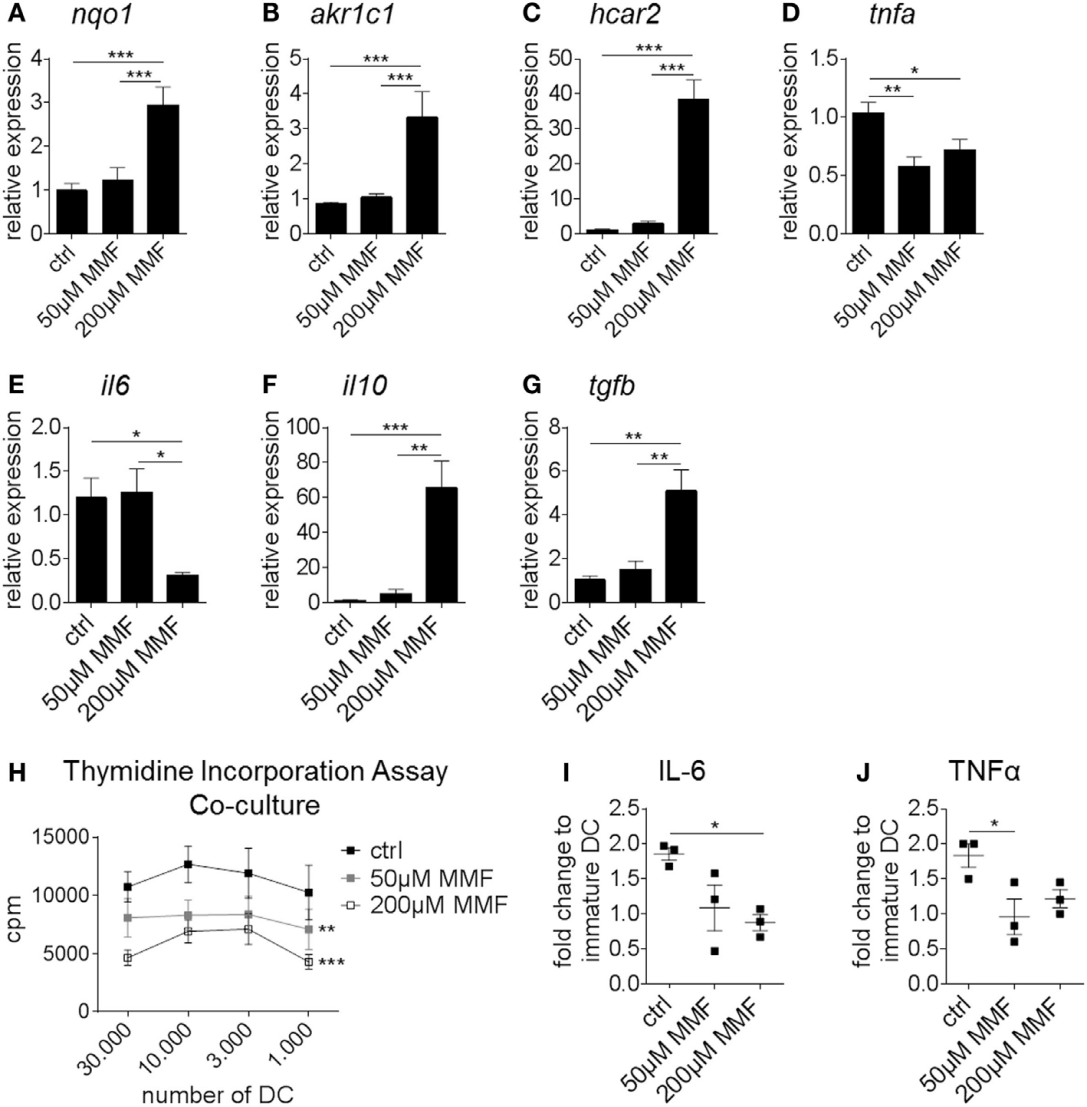
Nuclear factor (erythroid-derived 2)-like 2 (Nrf2) alters the expression profile and functionality of human dendritic cell (DC). **(A–G)** Gene expression analysis of *nqo1* **(A)**, *akr1c1* **(B)**, *hcar2* **(C)**, *tnfa* **(D)**, *il6* **(E)**, *il10* **(F)**, and *tgfb* **(G)** in untreated and metabolite monomethyl fumarate (MMF)-treated human monocyte-derived DC (data pooled from four preparations, *p < 0.05, **p < 0.01, ***p < 0.001). **(H)** Proliferation rate of allogeneic T cells cultured with MMF pretreated or untreated human monocyte-derived DC as assessed by thymidine incorporation assay (data pooled from four independent experiments, **p < 0.01, ***p < 0.001 compared to ctrl, cpm = counts per minute). **(I–J)** Interleukin (IL)-6 **(I)** and tumor necrosis factor alpha (TNFα) **(J)** secretion by MMF pretreated or untreated human monocyte-derived DC measured in the supernatant of T cell-DC coculture assays normalized to immature DC (data pooled from three independent experiments, *p < 0.05).

Expression analysis showed that Nrf2 activation enhanced the expression of *nqo1* and *aldo-keto reductase family 1 member C1 (akr1c1)* in human monocyte-derived DC analogously to murine BMDC (Figures 6A,B). Furthermore, MMF induced the expression of *hydroxycarboxylic acid receptor 2 (hcar2)*, a niacin receptor involved in the regulation of inflammation and oxidative stress independently from Nrf2 signaling (Figure 6C). Human DC-treated with MMF also exhibited reduced *tnfa* and *il6* but increased *il10* and *tgfb* expression compared to the control group (Figures 6D–G).

Thymidine incorporation revealed a robust reduction in the T cell proliferation rate when T cells were cultured together with MMF pretreated human monocyte-derived DC compared to untreated DC (Figure 6H). To verify our PCR results on the protein level, cytokine production was measured in the supernatant of the aforementioned coculture assays. Here, IL-6 (Figure 6I) and TNF-α (Figure 6J) secretion was significantly reduced when DC were pretreated with MMF.

Complementary to the murine model, induction of Nrf2 signaling in human monocyte-derived DC modulates maturation marker expression, cytokine production and DC induced T cell proliferation.

## Discussion

Here, we show that Nrf2 signaling plays a pivotal role for effects of fumarates on DC maturation, function and their capacity to regulate T cell responses in autoimmunity in mice and men. While DC specific Nrf2 deletion induces immunogenic DC with high costimulatory potential, activation of the Nrf2 signaling pathway *via* fumarates rather generates immature DC which show reduced proinflammatory gene and cytokine expression but enhanced tolerogenic properties also *in vivo*. Effects of fumarates on Nrf2 activation and on DC as well as a role of the Nrf2 pathway in murine and human DC have been described previously (15, 26, 29, 43, 44). Our study now links these observations and shows functional effects of fumaric acid esters on DC *via* the Nrf2 pathway as novel significance beyond what is already published.

In previous studies utilizing *in vitro* DC cultures, treatment with fumaric acid esters in murine and human systems retains DC in a rather immature, inactive state and reduces proinflammatory gene and cytokine expression (27, 28). We now extend these findings and show that fumaric acid esters activate the Nrf2 signaling pathway in DC thereby fostering anti-inflammatory properties in a time- and concentration-dependent manner. Furthermore, Nrf2 modulates DC-mediated T cell proliferation and differentiation specifically favoring the induction of regulatory T cells. The observed decrease in IL-12 production and enhanced secretion of IL-10 in our murine and human DC cultures after Nrf2 activation may be an explanation for the induction of anergy and tolerance in T cells (45).

Several groups employed Nrf2KO mice to demonstrate the relevance of Nrf2 signaling in DC biology *in vitro* (15, 17). We complement these data revealing that DC specific Nrf2 deletion generates strongly immunogenic DC that express high levels of costimulatory molecules. Nrf2-deficient DC also display marked migratory potential but low phagocytic capacity which renders them critical for stimulating peripheral T cell responses.

In MS and EAE as a prototype model for T cell-mediated autoimmunity, a beneficial effect of Nrf2 activation *via* DMF has already been shown (26, 46). Yet, these reports did not focus on DC and previous studies on the impact of fumaric acid esters on DC function mainly comprise *in vitro* data (28, 29). We utilized EAE in C57BL/6J mice as model to determine whether Nrf2 signaling in DC is also crucial for regulating autoreactive T cell responses *in vivo*. In the acute phase of EAE, the expression of costimulatory markers on CNS infiltrating DC is enhanced in Nrf2KO mice. Furthermore, injection of MMF pretreated DC shortly after EAE induction ameliorates the disease course and reduces the number of spinal cord infiltrating Th1 and Th17 cells. Therefore, the reported effects of fumarates on T cell populations may be rather indirect or in the case of lymphopenia even cytotoxic (32). Our data not only support the *in vitro* observation that Nrf2 induces immature, tolerogenic DC but also prove that the effect of Nrf2 signaling on DC function is critical enough to alter T cell activation and/or polarization in the CNS during neuroinflammation *in vivo*.

Interfering with costimulatory pathways has already been shown to effectively inhibit autoimmune diseases and allograft rejection (47). In the recent years, several molecular pathways, like NF-κB signaling (48), have been described as critically involved in DC maturation and function. These pathways are, however, difficult to modulate by pharmacological intervention since they are pivotally involved in a multitude of cellular functions and address various target structures. In contrast, the maturation status and phenotype of DC may be altered by weak Nrf2 inductors such as fumaric acid esters without causing serious side effects thereby creating a tolerogenic, anti-inflammatory environment (24, 26). Due to its role in managing oxidative stress responses, Nrf2 is not only involved in MS but also several other autoimmune diseases, such as psoriasis, asthma and diabetes, and different forms of cancer [as reviewed in Ref. (19, 49)]. In addition, Nrf2 plays a crucial role in neurodegeneration, cellular aging and senescence (50). Thus, the Nrf2 inducing effects of fumarates on antigen-presenting cells may not only be of interest in neuroinflammation, but also other diseases with a pivotal role of immune activation.

In conclusion, we demonstrate that fumaric acid esters modulate Nrf2 signaling in DC thus affecting their functionality in murine and human systems and leading to a more tolerogenic phenotype with the potential to ameliorate autoimmunity *in vivo*. In future studies, it will be of interest to determine DC-specific interaction partners of Nrf2 and the molecular pathways that are responsible for regulating the expression of cell surface molecules and the secretion of inflammatory and immunomodulatory cytokines.

## Ethics Statement

All experiments were performed in accordance with the German laws for animal protection and were approved by local ethic committees (Government of Unterfranken, Bavaria, Germany, ref. # 55.2-2532-2-451). For the generation of monocyte-derived DCs from leukoreduction system chambers of healthy donors, the positive vote from the local ethics committee at the University Erlangen-Nürnberg has been obtained (Re.-Nr.: 4556).

## Author Contributions

AnH, AW, IK, EZ, JB, SJ, KK, and CD planned and performed experiments and analyses. RL designed the study and planned as well as supervised the research. AnH, AW, and RAL wrote the manuscript. AS and JP provided genetic engineered mice. AS, RG, AiH, AB, JP, and DL supervised the research and edited the manuscript. All authors read and approved the final manuscript.

## Conflict of Interest Statement

The authors declare that the research was conducted in the absence of any commercial or financial relationships that could be construed as a potential conflict of interest.
