# Supplementary material for: Role of Nuclear Factor (Erythroid-Derived 2)-Like 2 Signaling for Effects of Fumaric Acid Esters on Dendritic Cells
Source: Front Immunol. 2017 Dec 22;8:1922. doi: 10.3389/fimmu.2017.01922 (PMC5744071; doi:10.3389/fimmu.2017.01922)
Supplement: Supplementary file 1 [file Data_Sheet_1.PDF]

Supplementary Material

Role of Nrf2 Signaling for Effects of Fumaric Acid Esters on Dendritic Cells

Anna Hammer MSc[1*], Anne Waschbisch MD[1*], Ilka Knippertz PhD[2], Elisabeth Zinser PhD[2], Johannes Berg MSc[3], Stefanie Jörg MSc[1], Kristina Kuhbandner MSc[1], Christina David BSc[3], Jingbo Pi MD, PhD[4], Antonios Bayas MD[5], De-Hyung Lee MD[1], Aiden Haghikia MD[3], Ralf Gold MD[3], Alexander Steinkasserer PhD[2] and Ralf A. Linker MD[1]

* equal contribution

Address correspondence to

Ralf Linker, Department of Neurology, Friedrich-Alexander-University Erlangen, Schwabachanlage 6, 91054 Erlangen, Germany, tel: +49-9131-85-32187, fax: +49-9131-85-34545, e-mail: ralf.linker@uk-erlangen.de

Supplementary figures

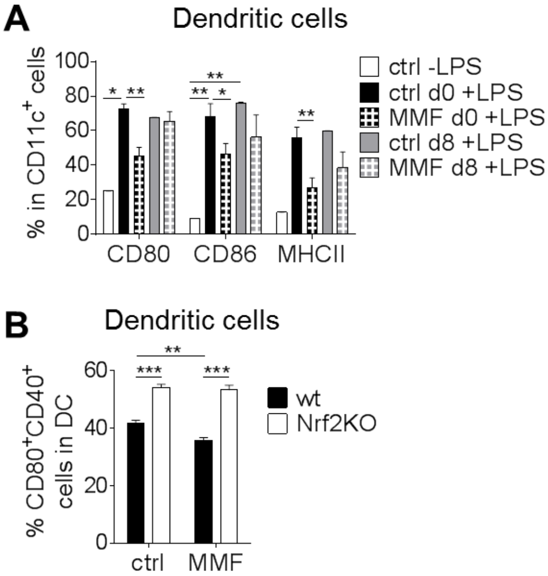

Suppl. Fig. 1: Nrf2 signaling modulates BMDC surface marker expression. (A) Expression of CD80, CD86 and MHCII on cultured CD11c^{+} BMDC as shown in Fig. 1B with additional controls: BMDC without LPS stimulation (ctrl -LPS) as immature control group; all other groups were matured by stimulation with 1 µg/ml LPS for 48h before analysis. For assessing the effect of MMF on BMDC maturation marker expression, 200 µM MMF in PBS were added to the cell culture medium either from the beginning (d0) or starting at day 8 together with LPS stimulation. Corresponding control cells were treated for the same time with only PBS (data pooled from three to four experiments, *$p<0.05$, **$p<0.01$). (B) Frequency of CD80^{+}CD40^{+} cells in CD11c^{+} BMDC generated from wt or Nrf2KO mice under MMF treatment as assessed by flow cytometry (data pooled from two experiments, **$p<0.01$, ***$p<0.001$). BMDC were stimulated with 1 µg/ml LPS for 48h before analysis.

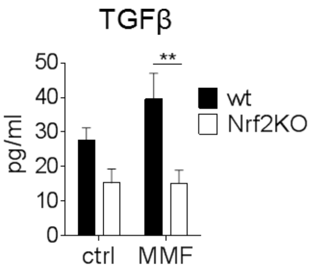

Suppl. Fig. 2: Nrf2 signaling induces TGFβ secretion in BMDC. TGFβ production by MMF treated wt and Nrf2-deficient cultured BMDC (n=4, **p<0.01).

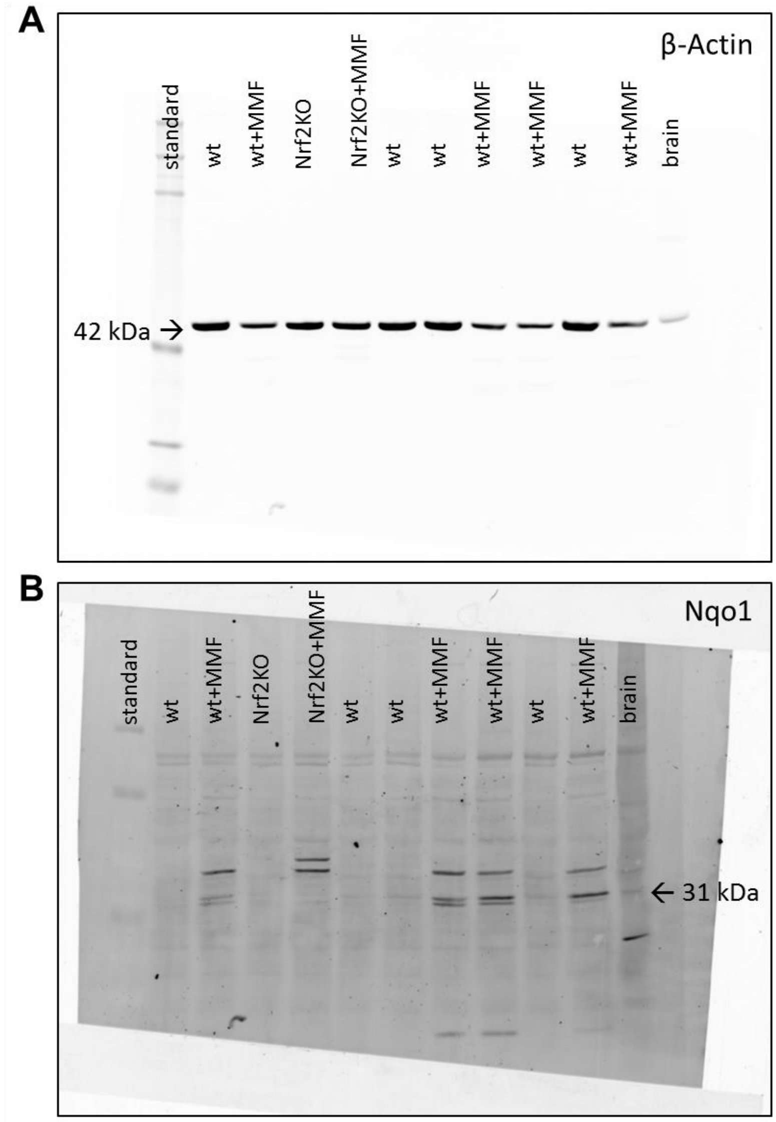

Suppl. Fig. 3: Nqo1 expression in murine BMDC. Original images of the western blots displayed in Fig. 2N.
